# Determining intestinal parasitic infections (IPIs) in inmates from Kajang Prison, Selangor, Malaysia for improved prison management

**DOI:** 10.1186/s12879-015-1178-3

**Published:** 2015-10-29

**Authors:** Lorainne Angal, Rohela Mahmud, Sajideh Samin, Nan-Jiun Yap, Romano Ngui, Amirah Amir, Init Ithoi, Adeeba Kamarulzaman, Yvonne AL Lim

**Affiliations:** Department of Parasitology, Faculty of Medicine, University of Malaya, Kuala Lumpur, Malaysia; Penjara Utama Kajang, Kajang, Selangor Malaysia; Department of Medicine, Faculty of Medicine, University of Malaya, Kuala Lumpur, Malaysia

**Keywords:** HIV, Intestinal parasites, Prison inmates, Malaysia

## Abstract

**Background:**

The prison management in Malaysia is proactively seeking to improve the health status of the prison inmates. Intestinal parasitic infections (IPIs) are widely distributed throughout the world and are still gaining great concern due to their significant morbidity and mortality among infected humans. In Malaysia, there is a paucity of information on IPIs among prison inmates. In order to further enhance the current health strategies employed, the present study aims to establish firm data on the prevalence and diversity of IPIs among HIV-infected and non-HIV-infected individuals in a prison, an area in which informed knowledge is still very limited.

**Methods:**

Samples were subjected to microscopy examination and serological test (only for *Strongyloides*). Speciation for parasites on microscopy-positive samples and seropositive samples for *Strongyloides* were further determined via polymerase chain reaction. SPSS was used for statistical analysis.

**Results:**

A total of 294 stool and blood samples each were successfully collected, involving 131 HIV positive and 163 HIV negative adult male inmates whose age ranged from 21 to 69-years-old. Overall prevalence showed 26.5 % was positive for various IPIs. The IPIs detected included *Blastocystis* sp., *Strongyloides stercoralis, Entamoeba* spp., *Cryptosporidium* spp., *Giardia* spp., and *Trichuris trichiura*. Comparatively, the rate of IPIs was slightly higher among the HIV positive inmates (27.5 %) than HIV negative inmates (25.8 %). Interestingly, seropositivity for *S. stercoralis* was more predominant in HIV negative inmates (10.4 %) compared to HIV-infected inmates (6.9 %), however these findings were not statistically significant. Polymerase chain reaction (PCR) confirmed the presence of *Blastocystis*, *Strongyloides*, *Entamoeba histolytica* and *E. dispar*.

**Conclusions:**

These data will enable the health care providers and prison management staff to understand the trend and epidemiological situations in HIV/parasitic co-infections in a prison. This information will further assist in providing evidence-based guidance to improve prevention, control and management strategies of IPIs co-infections among both HIV positive and HIV negative inmates in a prison environment.

## Background

Intestinal parasitic infections (IPIs) are highly transmissible in a crowded environment and in a closed-contact community such as in day-care centres and prisons. In Malaysia, high prevalence of parasitic infections among rural communities are commonly reported, however there is no available information on the occurrence of these infections among prison inmates. The only study among incarcerated population was conducted by Kamel *et al*. (1994) [1] on cryptosporidiosis among human immunodeficiency virus (HIV) positive drug addicts in Tampin Drug Rehabilitation Centre, Malaysia, reporting a prevalence of 23 %. Other foreign studies in Canada, Holland and Nigeria reported high prevalence of IPIs among prison inmates ranging from 27–77 % [2-5]. Ishaleku and Mamman (2014) [6] postulated that prison inmates have higher risk of acquiring IPIs due to the environment from which they come from and by the prison in which they live. Most professionals (e.g. clinicians and researchers) working in prison setup usually encountered problems such as lack of nutrition and concern, inadequate health care facilities and expertise which may further aggravate the spread of parasitic infections among prisoners.

Furthermore, prisoners also usually have high burden of HIV/AIDS. HIV prevalence among prisoners is between six to fifty times higher than that of the general adult population. Prisons have high prevalence of HIV-infected and at-risk populations, due to overcrowding, poor nutrition, limited access to health care, continued drug use, unsafe injecting practices, unprotected sex and tattooing. Furthermore, majority of the inmates come from marginalized populations with an elevated risk for HIV such as intravenous drug users (IVDUs) [7]. Both parasitic infections and HIV/AIDS are common problems present among prison inmates [8].

IPIs are known to be prevalent among the immunocompetent individuals in Malaysia [9-14]. However, the epidemiological status of these infections among immunocompromised people (i.e. HIV positive) is still scarce. Studies on correlation between cryptosporidiosis [1, 15-17], microsporidiosis [18], strongyloidiasis [19] and IPIs [20] among immunocompromised individuals (i.e. HIV-infected populations) are available. But most of the studies were among hospitalised individuals and one among the drug addicts. Currently, there are no studies on IPIs among the prison inmates in Malaysia.

The prison management in Malaysia is proactive in wanting to improve the health conditions of the prison inmates. Thus, the present study highlights the study of IPIs among the HIV-infected and non-HIV-infected inmates in a prison in the state of Selangor, Malaysia. Soil-transmitted helminths (STHs) including *Ascaris*, *Trichuris* and hookworms are the most common IPIs reported not only in Selangor but generally in Malaysia [21, 22]. It is important to determine and compare the IPIs between the HIV-infected and non-HIV-infected inmates because there is a high burden of HIV in the prison inmates and the parasitic infections among these two groups may differ.

In conjunction with that, the study aims to establish a platform to understand the trend and epidemiological situations in HIV positive, HIV negative and intestinal parasites co-infections in a prison setup. The prison management can consider for improved preventive and control strategies for the wellbeing of the inmates particularly pertaining to parasitic infections. Besides, the present study will help to contribute towards increase awareness among health care personnels, the prison inmates and public, the importance of providing guidance on better management of parasites co-infections in this population, and therefore help in reducing the adverse effects. It is also important to identify the associated factors which may favour the transmission of parasitic infections among the prison inmates in order to curb transmission.

## Methods

### Ethical approval and consent

Ethical approval was obtained prior to the commencement of this study from the Ethics Committee of the University of Malaya Medical Centre, Malaysia (UMMC; reference no. 890.10). Once approved, a briefing on the objectives and methodology of the study was presented to the Malaysian Prison Committee Board to obtain an official permission letter. Prior to sample collection, a briefing was carried out involving the researcher explaining the objectives and methodology of the study to the participants. Participants’ written consent were obtained before sampling commenced. The results of the present study have been submitted to the Kajang Prison authorities for further action. Treatment for positive cases will be administered by the authorized doctors in Kajang Prison.

### Sample size

The sample size required for this study was calculated based on the latest study on prevalence of IPIs among prison inmates [5] which is 77.0 % according to the formula by Leedy (1993) [23] as stated below. Taking into consideration a significance level of 5 % and confidence level of 95 %, a minimum sample size required for this study is 273. In the present study, 294 out of 314 inmates approached gave their consent to participate.

$$ \mathrm{n}=\left(\mathrm{z}/\mathrm{m}\right)2\mathrm{x}\mathrm{p}\left(1\hbox{-} \mathrm{p}\right) $$

*n* = sample size; z = standard core (1.96); m = rate of sampling error (5 %); *p* = estimated rate or case which happened in the population.

### Study area and population

The target population are the prison inmates who are either HIV positive or HIV negative regardless of their race and nationality. This study was carried out at the Kajang Prison, Selangor, Malaysia from June 2012 until January 2013 involving adult male inmates ranging from 21 to 70-years-old. Kajang Prison (3°0‘14“N 101°48’43”E) is situated next to the Prison Headquaters and is one of the 38 prison institutions under the administration of the Prison Department of Malaysia. It was established in 1975 and began full operation in 1985. It is one of the main prisons in Malaysia and was chosen as the study site due to its accessibility from University of Malaya (convenient for samples transportation) and high population of inmates. The prison management is very supportive of measures to further improve the wellbeing of their inmates. In general, the inmates at Kajang Prison encompass a majority of Malaysian citizens with a small composition of immigrants (i.e. Burmese and Indonesian).

The prison was divided into four divisions namely male division (also known as the main division), female division, treatment and drug rehabilitation division as well as the pre-free inmates division. At the time of samples collection, the male division accommodated an estimation of 3400 inmates, with approximately 5 % were infected with HIV. It can be further categorized into adults (21-years-old and above) and juveniles (below 21-years-old). Present study only involved the adult males.

Generally, there are about four to twelve inmates who are placed in the same cell. They are provided with basic facilities such as mattresses, latrine and tap water. Meals are given three times a day. Regimented cleaning schedule such as sweeping, mopping, washing all areas of the prison (e.g. own cells, kitchen, cafeteria) on daily basis is implemented for the inmates to keep the prison area clean. HIV-infected inmates are placed in one block, separately from non-HIV-infected inmates. It is an obligation for all inmates to undergo HIV test at the early stage of their sentence period.

### Questionnaires and samples collection

Following the participant’s written consent, questionnaires on demographic data, risks factors of having HIV, symptoms, disease history and condition in the prison’s cell was performed. During every collection visit, the researcher was accompanied by prison wardens who provided protection and assistance. CD4 count for HIV inmates was retrieved from their medical records at the prison clinic. Later, pre-labelled plastic stool containers were handed out to all participants. Over the next two days, filled stool containers were collected and blood-drawing session was carried-out. The fresh faecal samples (ambient temperature) and blood samples in plain bottles (placed in ice box) were transferred to the laboratory in the Department of Parasitology, Faculty of Medicine, University of Malaya within 2–4 h post-collection. In the present study, only one stool specimen was collected from each participant. Once the specimens arrived at the laboratory, a small portion of each fresh faecal sample was transferred into a 1.5 ml microcentrifuge tube respectively and the rest was preserved in 2.5 % potassium dichromate [24] and kept at 4 °C until further used. Blood samples in plain tubes were centrifuged at 5000 rpm for 10 min to obtain the serum. Serum was stored in −20 °C until further analysis. A total of 294 stool and blood samples each were successfully collected.

## Laboratory procedures

### Microscopy examination

Preserved faecal samples were processed by formalin ether concentration technique followed by iodine staining and were examined via microscopy (100× and 400× magnification) for the presence of intestinal protozoa and helminths. For detection and confirmation of *Cryptosporidium* spp., *Isospora belli* and *Cyclospora cayetanensis* oocysts, modified Ziehl-Neelsen staining was performed.

In addition, approximately 50 mg of the 294 fresh faecal samples were cultured into a 15-ml screw-cap tube containing 5 ml of Jones’ medium (consisting of sodium phosphate, potassium hydrogen phosphate, yeast extract and water) supplemented with 10 % horse serum [25] for the isolation of *Blastocystis* sp. The presence of *Blastocystis* sp. was observed daily for 14 days of cultivation, by placing 1 drop of the cultured sediment onto a glass slide, covered with a cover-slip and viewed (100× and 400× magnification) under light microscopy. Positive cultures were defined by the detection of any form of *Blastocystis* sp. (i.e. vacuolar, granular, amoeboid, and cystic forms) [26].

### Serological test of strongyloidiasis

Sera from all blood samples were subjected to *Strongyloides* ELISA Kit (Diagnostic Automation Inc., USA) for the qualitative detection of IgG antibodies toward *S. stecoralis*. The assay was performed according to the manufacturer’s instruction.

### Molecular characterization of microscopy-positive samples and seropositive samples

DNA was extracted from microscopy-positive and serology-positive faecal samples using Macherey Nagel NucleoSpin® Soil Kit (MACHEREY-NAGEL GmbH & Co. KG, Germany) according to the manufacturer’s protocol. Extracted samples were subjected to PCR according to the protocol for detection of respective species (Table 1). All PCR amplifications were done using MyCycler thermal cycler (Bio-Rad, Hercules, USA). Primers sequences, polymerase chain reaction (PCR) conditions and band size for respective species are tabulated in Table 1.

**Table 1:** Primers sequence and PCR conditions for molecular characterization of microscopy-positive and serology-positive samples

Species	PCR reaction	Primers set	Primers sequence (5’-3’)	PCR conditions					Total cycles (2–4)^f^	Product size	Reference
				1^a^	2^b^	3^c^	4^d^	5^e^			
*Blastocystis* sp.		Blast 505–532	F-GGA GGT AGT GAC AAT AAA TC	95 °C, 4 min	95 °C, 30 s	54 °C, 30 s	72 °C, 30 s	72 °C, 5 min	35	500 bp	Böhm-Gloning *et al*., 1997
		Blast 998–1017	R TGC TTT CGC ACT TGT TCA TC								Santin *et al*., 2011
*S. stercoralis*	Primary	SS–FO	F-ATC CTT CCA ATC GCT GTT GT	94 °C, 5 min	94 °C, 45 s	58 °C, 60 s	72 °C, 1 min	72 °C, 5 min	35		Nilforoushan *et al*., 2007
		SS–RO	R-TTT CGT GAT GGG CTA ATT CC								
	Secondary	SS–FI	F-GTA ACA AGG TTT TCG TAG GTG A	94 °C, 2 min	94 °C, 45 s	60 °C, 45 s	72 °C, 1 min	72 °C, 5 min	30	680 bp	
		SS–RI	R-ATT TAG TTT CTT TTC CTC CGC TT								
*Entamoeba*	Primary	E-1	F-TAA GAT GCA CGA GAG CGA AA	96 °C, 2 min	92 °C, 1 min	56 °C, 1 min	72 °C, 1 min 30 s	72 °C, 7 min	30		Khairnar & Parija, 2007
		E-2	R-GTA CAA AGG GCA GGG ACG TA								
*E. histolytica*	Secondary	EH-1	F-AAG CAT TGT TTC TAG ATC TGA G	96 °C, 2 min	92 °C, 1 min	48 °C, 1 min	72 °C, 1 min 30 s	72 °C, 7 min		439 bp	
		EH-2	R-AAG AGG TCT AAC CGA AAT TAG								
*E.dispar*		ED-1	F-TCT AAT TTC GAT TAG AAC TCT							174 bp	
		ED-2	R-TCC CTA CCTATT AGA CAT AGC								
*E. moshkovskii*		Mos-1	F-GAA ACC AAG AGT TTC ACA AC							553 bp	
		Mos-2	R-CAA TAT AAG GCT TGG ATG AT								
											Reference
								5			
*C. parvum*	Primary	N-DIAGF2	F-CAA TTG GAG GGC AAG TCT GGT GCC AGC	95 °C, 5 min	94 °C, 30 sec	68 °C, 1 min	72 °C, 30 sec	72 °C, 10 min	35	655 to 667 bp	Nichols *et al*., 2003
		N-DIAGR2	R-CCT TCC TAT GTC TGG ACC TGG TGA GT								
	Secondary	CPB-DIAGF	F-AAG CTC GTA GTA GTT GGA TTC TG	95 °C, 5 min	94 °C, 30 sec	60 °C, 1 min	72 °C, 30 sec	72 °C, 10 min		435 bp	Johnson *et al*., 1995
		CPB-DIAGR	R-TAA GGT GCT GAA GGA GTA AGG								

^a^Initial denaturation

^b^Denaturation

^c^Annealing

^d^Extension

^e^Final extension

^f^Total cycles only applicable for PCR conditions 2–4

Microscopy-positive samples for *Blastocystis* sp. were subjected to single-step PCR protocol for specific amplification of *Blastocystis* sp*.* [27, 28]. Nested multiplex PCR targeting a 16S-like rRNA gene was carried out on positive faecal samples for molecular characterization of *E. histolytica, E.dispar* and *E. moshkovskii* [29]. Primary PCR was performed for amplification of *Entamoeba* genus in the positive faecal samples. Subsequently, primary PCR product was subjected to secondary PCR reaction for species-specific identification. Nested PCR protocol was performed targeting the SSU rRNA gene for the detection of *Cryptosporidium* in the microscopy-positive samples [30, 31]. Seropositive samples for strongyloidasis were subjected to nested PCR targeting the internal transcribed spacer 1 (ITS1) region of the ribosomal DNA gene [32].

In all the PCR reaction, positive controls were used according to the species studied and distilled water was used as negative controls. PCR products were analyzed on 1 % agarose gel (*Blastocystis* sp.) and 2 % agarose gel (*Entamoeba* spp., *C. parvum* and *S. stercoralis*) visualized using UV transiluminator after staining with SYBR® Safe DNA (Invitrogen, Auckland, New Zealand). PCR-positive samples for *Blastocystis* sp. were sent for sequencing and analyzed with BLAST (*Basic Local Alignment Search Tool)* for further determination of *Blastocystis* subtypes.

### Data analysis

SPSS software (Statistical Package for the Social Sciences) program for Windows version 22 (SPSS, Chicago, IL, USA) was employed for data entry and statistical analysis. Descriptive statistics were mainly used to describe the characteristics of the study population including prevalence of the IPIs. Qualitative data were determined and presented as frequencies and percentages. Statistical significance of differences in proportions was evaluated by Chi-Square test with significant value of *p* < 0.05 used for all tests. Univariate analysis was run to determine the association of variables with IPIs infections.

## Results

### Demographic distributions and clinical information of participating prison inmates

From a total of 294 consented inmates, there were 131 (44.6 %) HIV positive inmates and 163 (55.4 %) HIV negative inmates. The inmates aged ranging from 20 to 69-years-old. Participants included Malay, Chinese, Indian, Sabah and Sarawak Bumiputera ethnics, with a small composition of immigrants (i.e. Burmese and Indonesian). Majority of them had secondary education. Symptoms related to IPIs and other diseases presences were also noted.

Intravenous drug users (IVDUs) were identified to be the main mode of HIV transmission among the 131 HIV positive inmates. Duration of having HIV ranged from 0 to more than 21 years. As some of the HIV positive inmates’ medical files could not be retrieved from the record section, CD4 counts were only available for 93 out of the 131 HIV positive inmates, with 24 (25.8 % of 93) having CD4 counts < 200 cells/mm^3^ and 69 (64.2 %) with CD4 counts > 200 cells/mm^3^. All HIV positive inmates were offered to be included in the highly active antiretroviral therapy (HAART) programme by the prison’s clinic. However, there were only 7 (5.3 %) who were willing to undergo HAART due to reasons such as side effects and non-compliance. Table 2 shows the details of demographic distributions and clinical information of participating prison inmates according to HIV status.

**Table 2:** Demographic and clinical information of participating prison inmates according to HIV status

Characteristics	Overall(*n* = 294)		HIV positive (*n* = 131; 44.6 %)		HIV negative (*n* = 163; 55.4 %)	
	No.	%	No.	%	No.	%
Age group (years)						
20-29	67	22.8	17	13.0	50	30.7
30-39	115	39.1	55	42.0	60	36.8
40-49	80	27.2	49	37.4	31	19.0
50-59	26	8.8	8	6.1	18	11.0
60-69	6	2.0	2	1.5	4	2.5
Ethnicity						
Malay	171	58.2	84	64.1	87	53.4
Chinese	44	15.0	14	10.7	30	18.4
Indian	62	21.1	26	19.8	36	22.1
Sabah Bumiputera	8	2.7	1	0.8	7	4.3
Sarawak Bumiputera	2	0.7	1	0.8	1	0.6
Immigrants	7	2.4	5	3.8	2	1.2
Duration in prison (months)						
0-3	161	54.8	71	54.2	90	55.2
4-6	59	20.1	12	9.2	47	28.8
7-9	26	8.8	5	3.8	21	12.9
10-12	7	2.8	4	3.1	3	1.8
>12	41	13.9	39	29.8	2	1.2
Education attainment						
No formal education	10	3.4	8	6.1	2	1.2
Primary school	72	24.5	27	20.6	45	27.6
Secondary school	202	68.7	94	71.8	108	66.3
Tertiary school	10	3.4	2	1.5	8	4.9
Symptoms related to GIT^a^ infection						
Diarrhoea	36	12.2	21	16.0	15	9.2
Nausea and vomiting	16	5.4	12	9.2	4	2.5
Abdominal pain	45	15.3	28	21.4	17	10.4
Dysentery	27	9.2	22	16.8	5	3.1
Feeling tired easily	111	37.8	69	52.7	42	25.8
Fever	64	21.8	35	26.7	29	17.8
Loss of appetite	29	9.9	19	14.5	10	6.1
Loss of weight	41	13.9	17	13.0	24	14.7
Other diseases present						
Tuberculosis	19	6.5	18	13.7	1	0.6
Hepatitis B or C	22	7.5	18	13.7	4	2.5
Asthma	13	4.4	8	6.1	5	3.1
Hypertension	3	1.0	0	0	3	1.8
Diabetes	11	3.7	1	0.8	10	6.1
Schizophrenia	6	2.0	1	0.8	5	3.1
Mode of HIV transmission						
IVDU^b^			86	65.6		
Sexual transmission			11	8.4		
Both			24	18.3		
Unknown			10	7.6		
Duration of having HIV (years)						
0-5			55	42.0		
6-10			32	24.4		
11-15			28	21.4		
16-20			15	11.5		
>21			1	0.8		
HAART^c^						
Yes			55	42.0		
No			32	24.4		
CD4 count (cells/mm^3^)						
<200			24	18.3		
>200			69	52.7		

^a^ *GIT* Gastrointestinal tract

^b^ *IVDU* Intravenous drug user

^c^ *HAART* Highly active antiretroviral therapy

### Intestinal parasitic infections (IPIs) among prison inmates

Table 3 shows the overall prevalence of IPIs among the 294 studied inmates was 26.5 % (78/294). Intestinal parasites detected included *Blastocystis* sp. in 43 inmates (14.6 %), *S. stercoralis* with seropositivity in 26 inmates (8.8 %), *Entamoeba* spp. in 8 inmates (2.7 %), *Cryptosporidium* spp. in 7 inmates (2.4 %) and both *Giardia* spp. and *T. trichiura* in only 1 inmate each (0.3 %). Majority of them, 23.8 % (70/294) had single infections and there was 2.7 % (8/294) who were infected with two parasites.

**Table 3:** Distribution of parasitic infections in HIV positive and HIV negative prison inmates

Species	Overall (*n* = 294)		95 % CI^a^	HIV positive (*n* = 131)		95 % CI	HIV negative (*n* = 163)		95 % CI	*p*
	No.	%		No.	%		No.	%		
Overall	78	26.5	21.81-31.86	36	27.5	20.56-35.68	42	25.8	19.67-32.98	0.741
*Blastocystis* sp.	43	14.6	11.04-19.12	16	12.2	7.66-18.92	27	16.6	11.64-23.03	0.294
*S. stercoralis*	26	8.8	6.11-12.64	9	6.9	3.66-12.54	17	10.4	6.61-16.07	0.285
*Entamoeba* spp.	8	2.7	1.39-5.28	6	4.6	2.12-9.63	2	1.2	0.34-4.36	0.145^b^
*Cryptosporidium* spp.	7	2.4	1.16-4.83	5	3.8	1.64-8.62	2	1.2	0.34-4.36	0.248^b^
*Giardia* spp.	1	0.3	0.06-1.90	1	0.8	0.13-4.20	0	0	0	0.466^b^
*T. trichiura*	1	0.3	0.06-0.190	1	0.8	0.13-4.20	0	0	0	0.446^b^
Single infection	70	23.8	19.30-29.00	34	26.0	19.21-34.07	36	22.1	16.40-29.06	0.439
Double infections	8	2.7	1.39-5.28	2	1.5	0.42-5.40	6	3.7	1.70-7.80	0.306^b^

^a^ *CI* Confidence interval

^b^tested by the Fisher exact test, others were tested by the chi-square test

With regards to IPIs according to HIV status, the inmates who were HIV positive had slightly higher rates of IPIs with 27.5 % (36/131) compared to HIV negative inmates with 25.8 % (42/163), however it was not statistically significant (*p* = 0.741). Higher prevalence of both *Entamoeba* spp., 4.6 % (6/131; *p* = 0.145) and *Cryptosporidium* spp., 3.8 % (5/131; *p* = 0.248) were seen in HIV positive inmates. Similar prevalence of *Giardia* spp., 0.8 % (1/131) and *Trichuris trichiura*, 0.8 % (1/131) were also observed among this group. In contrast, *Blastocystis* sp. infection, 16.6 % (27/163; *p* = 0.294) was slightly higher in HIV negative inmates compared to HIV positive inmates with 12.2 % (16/131). Interestingly, seropositive of *S. stercoralis* was higher in HIV negative inmates, 10.4 % (17/163; *p* = 0.285) compared to HIV positive inmates, 6.9 % (9/131).

### Risk factors for IPIs in prison inmates

Univariate analysis for the association of parasitic infections with sociodemographic characteristics (i.e. age group, education attainment), presence of tuberculosis and presence of hepatitis B or C among the overall inmates, HIV positive inmates and HIV negative inmates are shown in Table 4. Besides that, factors including duration of having HIV, HAART treatment, and CD4 count were also analysed in HIV positive inmates. Only those co-infected with tuberculosis appeared to have higher odds (i.e., 2.13) in getting IPIs compared to those without among the overall inmates. The same trends were observed in which those co-infected with tuberculosis (OR = 2.43), those with CD4 count < 200 cells/mm (OR = 1.32) and those having HIV less than 10 years (OR = 1.45) have higher odds in getting IPIs in HIV-infected inmates. In HIV negative inmates, the trends observed were those within age group of 40-years-old and above (OR = 1.21) and those co-infected with hepatitis B or C (OR = 2.98) have higher odds in getting IPIs. However, no variables were found to be significantly associated with IPIs in the studied population (*p* > 0.05).

**Table 4:** Risk factors of intestinal parasitic infections among participating prison inmates according to HIV status (n = 294)

Characteristics	Overall (*n* = 294)			HIV positive (*n* = 131)			HIV negative (*n* = 163)		
	No. infected (%)	OR (95 % CI)	*p*	No. infected (%)	OR^a^ (95 % CI)	*p*	No. infected (%)	OR (95 % CI)	*p*
Age group (years)									
≥40	31 (27.7)	1.10 (0.65-1.87)	0.727	16 (27.1)	0.97 (0.45-2.09)	0.933	15 (28.3)	1.21 (0.58-2.54)	0.607
<40	47 (25.8)	1		20 (27.8)	1^b^		27 (24.5)	1	
Education attainment									
Primary school and below	20 (24.4)	0.86 (0.48-1.54)	0.605	7 (20.0)	0.58 (0.23-1.47)	0.247	13 (27.7)	1.15 (0.53-2.47)	0.725
Secondary school and below	58 (27.4)	1		29 (30.2)	1		29 (25.0)	1	
Tuberculosis									
Yes	8 (42.1)	2.13 (0.82-5.51)	0.112	8 (44.4)	2.43 (0.87-6.76)	0.094^e^	0	NA^f^	0.535^e^
No	70 (25.5)	1		28 (24.8)	1		42 (25.9)		
Hepatitis B or C									
Yes	4 (18.2)	0.60 (0.20-1.82)	0.356	2 (11.1)	0.29 (0.06-1.33)	0.153^e^	2 (50.0)	2.98 (0.41-21.82)	0.274^e^
No	74 (27.2)	1		34 (30.1)	1		40 (25.2)	1	
CD4 count^c^ (cells/mm^3^)									
≤200				8 (33.3)	1.32 (0.48-3.58)	0.590			
>200				19 (27.5)	1				
Duration of having HIV (years)									
≤10				26 (29.9)	1.43 (0.63-3.36)	0.386			
>10				10 (22.7)	1				
HAART^d^									
No				32 (26.9)	0.74 (0.21-2.61)	0.736^e^			
Yes				4 (33.3)	1				

^a^ *OR* Odd ratio

^b^Reference group marked as OR = 1

^c^ *n* = 93

^d^ *HAART* Highly active antiretroviral therapy

^e^Fisher’s exact value

^f^ *NA* Not available

### Molecular characterization of *Blastocystis*, *Entamoeba*, *Cryptosporidium* and *Strongyloides* species

Out of 43 microscopy-positive samples for *Blastocystis* sp. that were subjected to PCR, 42 samples (97.7 %) were successfully amplified and confirmed as *Blastocystis* sp. Three out of 26 serology-positive samples (11.54 %) were positive for *S. stercoralis*. Molecular detection of *Entamoeba* spp. showed only 1 out of 8 (12.5 %) *Entamoeba* microscopy-positive samples was positive for both *E. histolytica* and *E. dispar*. However, none of the *Cryptosporidium* microscopy-positive samples were successfully amplified using the PCR method.

### *Blastocystis* sp. subtype determination via DNA sequencing and BLAST analysis

There were 33 out of 42 samples which had been successfully sequenced. BLAST results of the 33 sequences showed that the inmates harboured 3 subtypes of *Blastocystis* sp*.* namely subtype 1 (7/33; 21.2 %), subtype 3 (25/33; 75.8 %), and subtype 6 (1/33; 3.0 %). Another 9 samples were not sequenced due to insufficient DNA concentration.

## Discussion

The present study reported a total prevalence of 26.5 % (78 out of 294) of IPIs among the prison inmates. Comparatively, the prevalence is lower from previous studies conducted in prison inmates in other countries such as in Canada, 27.16 % [2] and Africa with over 60 % [4, 5, 33]. Better infrastructure and sanitation provided by the prison management and increase health consciousness among prisoners in Malaysia could have contributed to this apparent lower prevalence. *Blastocystis* sp. was the most common parasite found in the present study, in contrast with previous studies with STHs, *Giardia* spp and *Entamoeba* spp. were mostly found. Previous studies did not perform test to detect *Blastocytis* sp., thus the prevalence for this parasite is not known.

The present study is the first epidemiological study on parasitic infections among prison inmates in Malaysia. Thus, it is not possible to do any comparison locally. A local study on cryptosporidiosis among inmates in drug rehabilitation centre, which can be considered to have almost similar setting with prison was attempted by Kamel *et al*. (1994) [1]. A prevalence of 23 % was recorded from this study. However, the IPIs prevalence in the present study is higher in comparison with the general population [34, 35]. Nonetheless, the IPIs prevalence in the present study is lower compared to the IPIs prevalence among the Orang Asli (indigenous) population based on reports from various studies [21, 36].

Parasitic infections among HIV positive individuals are increasing rapidly especially among those incarcerated populations such as prisoners. Majority of the previous studies reported higher prevalence of intestinal parasites among those infected with HIV compared to those who were not. Although in the present study, the prevalence of IPIs in HIV positive inmates was found to be slightly higher (27.5 %; 36 out of 131) than in HIV negative inmates (25.8 %; 42 out of 163), it was not statistically significant. HIV positive status of some of the prisoners did not play a major role in increasing the IPIs prevalence.

Presence of *Blastocystis* sp. in the present study was higher in HIV negative inmates than HIV positive inmates with 16.6 % (27/163) and 12.2 % (16/131), respectively. Though many authors have given credit to it as a pathogen, however there is still much controversy surrounding the role of *Blastocystis* in causing human disease. The most common symptoms associated with *Blastocystis* infection include diarrhoea, abdominal pain and vomiting. The significance of *Blastocystis* sp.in HIV/AIDS populations has not been ascertained, but there have been several studies with varying results on the prevalence of intestinal parasites and particularly the incidence of *Blastocystis* in these populations [37, 38]. Based on these previous studies, *Blastocystis* infection may have a significant role in causing diarrhoea especially in the immunosuppressed patients.

In the present study, *Blastocystis* sp. subtype 3 was found to be the most common subtype, a finding in agreement with the previous studies [39, 40]. Occurrence of *Blastocystis* sp. subtype 1 (ST1) following ST3 was not surprising. There were various reports which showed ST1 was the common subtype found in human, together with ST2, ST3 and ST4 [39, 41, 42]. The single infection of *Blastocystis* subtype 6 (ST6) may reflect zoonotic transmission, as this subtype has been reported among the avians [43, 44]. Jantermtor *et al*. (2013) [40] also detected ST6 among the Thais in their study. However, ST6 is rarely reported in Asia.

*S. stercoralis* was negative microscopically in the present study. This may be due to a one stool sample collection from each participant. Multiple stool specimens are required for microscopy diagnosis of *S. strongyloides* because the parasite is excreted periodically and in low numbers. In the present study, ELISA test showed high seroprevalence of *S. stercoralis* in HIV negative inmates (10.4 %; 17/163) compared to in HIV positive inmates (6.9 %; 9/131). This trend may be due to the inmate’s geographical origin, hygiene and occupation before being imprisoned. There were also a few studies which reported low prevalence of *S. stercoralis* in HIV positive individuals [45, 46]. However, ELISA test is unable to differentiate current from past infection. Based on PCR of stool samples, *S. stercoralis* was detected in 3 non-HIV inmates. Since PCR analysis confirmed current infection, these positive individuals should be treated to prevent transmission and hyperinfection.

Opportunistic infection of *Cryptosporidium* spp. was more predominant (3.8 %; 5/131) in HIV positive inmates than in non-HIV inmates (1.2 %; 2/163), which was in concordance with the study by Tian *et al*. (2012) [47]. Cryptosporidiosis is categorized by CDC as AIDS-defining illness (ADI) due to its ability in causing diarrhoea and poses a public health problem in HIV/AIDS patients [48]. In clinical cryptosporidiosis, chronic diarrhoea with watery stools, weight loss and dehydration are the prominent features in symptomatic patients [49]. Seven (7) inmates were microscopically positive for *Cryptosporidium* spp. Detection of *Cryptopsoridium* by Ziehl-Neelsen stain is a definitive diagnosis. Of these, none of the samples were successfully amplified using nested PCR [31]. These microscopy-positive samples failed to be detected maybe due to low DNA concentration of the parasites or degradation of DNA.The present study reported *Entamoeba* spp. was more prevalent among the HIV positive inmates (4.6 %; 6/131) in comparison with the non-HIV inmates (1.2 %; 2/163) microscopically. This finding was in concordance with the previous study [38]. However, *E. histolytica* cannot be differentiated microscopically with the other two non-pathogenic species namely *E. dispar* and *E. moshkovskii* due to similarity in their morphology. Thus, PCR test was performed in order to distinguish the pathogenic from non-pathogenic species.

Among all the intestinal parasites reported in the present study, *S. stercoralis* and *T. trichiura* are categorized as soil-transmitted helminths (STHs). Thus, to prevent the infection of these parasites, (i) regular anthelminthic treatment, (ii) health education, (iii) sanitation and personal hygiene can be implemented. However, in the present study setting, prevalence of STHs is low. This may be due to the good practice of sanitation and personal hygiene by the inmates.

With regards to CD4 counts, those who had CD4 counts < 200 cells/mm^3^ were having higher odds in getting IPIs compared to those with CD4 counts > 200 cells/mm^3^. These findings were consistent with other study that showed parasitic infections usually occurred in individuals having CD4 counts of < 200 cells/mm^3^ [20]. Furthermore, those newly acquired HIV infection between 0 to 10 years had higher IPIs compared to those who acquired HIV infection >10 years. Inmate’s lack of information on hygiene, sanitation and of the infection could be the reasons for this outcome. In addition, majority of this group of inmates were also not on HAART. Those HIV infected inmates who were undergoing HAART have lower levels of IPIs in comparison to those who were not. Hung *et al*. (2007) [50] addressed the importance of HAART among HIV positive individuals in restoring their immune functions, thus improving the immunity level towards IPIs.

Tuberculosis (TB) and hepatitis B or C (hep B or C) are the most common co-infections among HIV positive people worldwide. WHO (2015) [51] estimated at least one-third of people living with HIV worldwide in 2013 were infected with TB whereas almost 80 % of people with HIV who inject drugs also have hepatitis B or C virus [52]. Among the HIV negative inmates, those co-infected with hep B or C had higher probability of acquiring IPIs compared to those without. On the other hand, among overall inmates, those co-infected with TB have higher odds in getting IPIs in comparison to those without TB regardless of their HIV status. This affirmed further that TB and hep B or C worsened the immunity level, thus increasing the individual’s susceptibility towards parasitic infections in this study.

Few limitations were encountered in the present study including unretrievable CD4 count for some of the inmates as their clinical records were not found in the record section; conditions of the prison cells were not observed as these areas were restricted only for the inmates, staffs and doctors in-charge and comparison on parasitic infections with the female inmates could not be done as permission to take samples was only given for the male inmates.

## Conclusion

Overall prevalence of IPIs among inmates was 26.5 % (78 out of 294). With regards to the HIV status, HIV positive inmates had slightly higher rates of IPIs with 27.5 % compared to HIV negative inmates with 25.8 %. The present study is the first study in Malaysia which emphasized on the parasites-HIV co-infections in the prison setting. Non-HIV inmates were also included to compare the prevalence of parasites between these two groups. It was noted in the present study that IPIs prevalence is lower compared to previous studies on prison inmates conducted in other countries. Malaysian Prison Department is proactively improving the health status of prison inmates in Malaysia. The information gathered from the study will enable the health care providers and prison management staff to understand the trend and epidemiological situations of parasitic co-infections in a prison setup. It also provides evidence-based guidance to improve prevention and control (i.e. food preparation, boiled drinking water) through health education as well as treatment management strategies of IPIs co-infections (i.e. parasite infections screening, anti-parasitic treatments). More importantly, the inmates themselves should be aware of the importance of good personal hygiene to prevent them from acquiring or transmitting infections to others. In addition, doctors managing these infected individuals should also have heightened awareness of the possibility of co-infections with parasites, bacteria and viruses.

## Abbreviations

AIDS: Acquired immunodeficiency syndrome
BLAST: Basic local alignment search tool
CI: Confidence interval
DNA: Deoxyribonucleic acid
GIT: Gastrointestinal tract
HAART: Highly active antiretroviral therapy
Hep B or C: Hepatitis B or C
HIV: Human immunodeficiency virus
IPIs: Intestinal parasitic infections
IVDUs: Intravenous drug users
OR: Odds ratio
PCR: Polymerase chain reaction
Spp: Species
SPSS: Statistical Package for the Social Sciences
SSU rDNA: Small subunit ribosomal DNA
ST: Subtype
STI: Sexually transmitted infections
STHs: Soil-transmitted helminths
TB: Tuberculosis
UMMC: University Malaya Medical Centre
UNAIDS: United Nations & AIDS
WHO: World Health Organization
